# The Mental Health and Social Media Use of Young Australians during the COVID-19 Pandemic

**DOI:** 10.3390/ijerph19031077

**Published:** 2022-01-19

**Authors:** Eleanor Bailey, Alexandra Boland, Imogen Bell, Jennifer Nicholas, Louise La Sala, Jo Robinson

**Affiliations:** 1 Orygen, Parkville, VIC 3052, Australia; imogen.bell@orygen.org.au (I.B.); jennifer.nicholas@orygen.org.au (J.N.); louise.lasala@orygen.org.au (L.L.S.); jo.robinson@orygen.org.au (J.R.); 2 Centre for Youth Mental Health, University of Melbourne, Parkville, VIC 3010, Australia; 3 Department of Family Medicine & Community Health, University of Massachusetts Medical School, Worcester, MA 01655, USA; aboland936@yahoo.com

**Keywords:** COVID-19, social media, youth, mental health, self-harm, suicide, self-harm, digital technology

## Abstract

Young people may be particularly vulnerable to the mental health impacts of the COVID-19 pandemic and may also be more likely to use social media at this time. This study aimed to explore young people’s mental health and social media use during the COVID-19 pandemic and examined their use of social media to seek and provide support for suicidal thoughts and self-harm during this period. Young people aged 16–25 (*n* = 371, M = 21.1) from the general population in Australia completed an anonymous, cross-sectional online survey advertised on social media from June to October 2020. Participants reported high levels of psychological distress, with over 40% reporting severe levels of anxiety and depression, and those with a mental health diagnosis were more likely to perceive the pandemic to have had a negative impact on their mental health. Gender-diverse participants appeared the most negatively impacted. Social media use was high, with 96% reporting use at least once a day, and two-thirds reporting an increase in social media use since the start of the pandemic. One-third had used social media to seek support for suicidal thoughts or self-harm, and half had used it to support another person. This study adds to a growing literature suggesting social media can provide an opportunity to support young people experiencing psychological distress and suicide risk. Uniquely, this study points to the utility of using social media for this purpose during high-risk periods such as pandemics, where access to face-to-face support may be limited. To promote the quality and safety of support provided on social media, resources for help-seekers and help-givers should be developed and disseminated. Social media companies must consider the vulnerability of some users during pandemics and do what they can to promote wellbeing and safety.

## 1. Introduction

The COVID-19 pandemic and associated response have led to unprecedented disruptions to daily life. Documented stressors associated with worldwide pandemics, including the COVID-19 pandemic, include a reduced sense of control, heightened uncertainty, worries about health and the health of loved ones, economic and lifestyle disruptions, and increased isolation and loneliness [1]. As such, the potential impact of the COVID-19 pandemic on mental health has been a major focus of attention. Several systematic reviews and meta-analyses have confirmed the association of the COVID-19 pandemic with high levels of psychological distress, depression, and anxiety [2,3,4], with some research suggesting individuals with underlying mental health problems are at higher risk of poor mental health outcomes [5]. Young people may experience unique challenges associated with COVID-19, rendering them particularly vulnerable to poor mental health outcomes. For example, many countries have closed schools and universities in favour of online learning, leading not only to a disrupted education, but also to a reduced opportunity to form and maintain social relationships [6]. Similarly, young people are over-represented in many of the industries hardest hit by the COVID-19 lockdown measures, such as retail and hospitality [7]. In addition to a disrupted routine, reduced access to coping strategies and limited access to social networks, for some young people, increased time at home means an increased risk of exposure to maltreatment and family violence [8,9,10]. It is therefore unsurprising that studies specifically examining the association between COVID-19 and the mental health outcomes in adolescents and young people have found that this age group experiences high levels of psychological distress [4,11,12,13]. Young people, particularly young females, may also be at an elevated risk of self-harm and suicide [14,15,16,17].

In addition to being at an elevated risk of adverse mental health outcomes, young people may also be less likely to access professional mental health treatment. Studies have shown that the rates of professional help-seeking by young people for mental health problems, including suicidal thoughts and self-harm, are generally low [18], and some research shows they may be even lower as a result of COVID-19 lockdown measures [19]. In general, young people often prefer to seek support and information about mental health problems from informal sources, such as friends, the internet, and/or via social media [18]. Our previous research has shown that social media is often used to seek help for emotional problems, including suicide [20]. Therefore, given that social media use overall has increased during the COVID-19 pandemic [21], and young people are heavy users of social media in general [22], it is likely that young people’s social media use, including for help-seeking and mental health support, has increased during the pandemic. While social media may have benefits related to mental health and suicide prevention, including enabling users to express their emotions in a non-judgmental forum, the facilitation of mutual social support, and promotion of engagement and retention in mental health treatment [20,23], risks include reduced face-to-face social engagement, a negative impact on daily activities, and exposure to hostile or harmful content such as bullying or harassment [23]. Additional risks associated with the use of social media to seek support for suicidal thoughts or self-harm include the potential for exposure to “triggering” or distressing content, and relatedly, the potential for contagion of suicidal behaviour to occur [24]. Importantly, if young people use social media at higher levels during pandemics, at a time where their distress and suicide risk may also be elevated, then social media may also provide an opportunity to deliver material designed to educate and/or support these young people.

Taken together, the COVID-19 pandemic is expected to have negative consequences for the mental health of young people. It may also lead them to spend more time online, with increased use of social media for seeking help for mental health problems, suicidal thoughts, and/or self-harm. The aim of the present study was to examine mental health and social media use, including the use of social media relating to suicidal thoughts or self-harm, in a community-based sample of Australian young people during the COVID-19 pandemic.

## 2. Methods

### 2.1. Setting and Design

This study involved a cross-sectional anonymous community survey advertised via social media to young people across Australia. The study was conducted by researchers at Orygen, a youth mental health research and translation organisation based in Melbourne, Australia. A survey was disseminated during the months of June through to October 2020, coinciding with Australian Federal and State government-mandated lockdown restrictions (“stage 3”) in which all Australians were required to ‘socially isolate’ from anyone outside their primary residence, and limited to leaving their residence for only essential activities, defined as: employment; providing care, including seeking medical care; exercise; and to shop for food and other necessary goods/services. The survey formed part of a larger study (“BRACE”) which involved a survey to examine the effect of COVID-19 on the mental health and wellbeing of young Australians, the impact of the COVID-19 pandemic on primary mental health service delivery, and the potential role of technology in supporting young people’s mental health. Data reported here are derived from the general population survey, which included measures of the impact of COVID-19 on mental health and wellbeing, social media use, and the use of and attitudes towards technologies. The focus of this paper is on mental health and social media use data collected from the community-based sample.

### 2.2. Participants and Procedure

Young people were eligible for participation if they were aged between 16 and 25 years (inclusive) and lived in Australia. There were no other exclusion criteria. Since convenience sampling was used to recruit participants, no target sample size was specified; rather, the aim was to recruit as large a sample as possible within the 5-month recruitment period.

Participants were recruited via targeted advertisements on Facebook and Instagram posts from June to October 2020. The study was advertised to young people from all states across Australia from June to August, with targeted advertising for those in Victoria from August to October due to continuing local lockdown laws occurring only in this region during this time. The brief social media advertisement included a link forwarding potential participants to a web page containing more detailed information. From there, participants could follow a link to the full participant information and consent form, after which they completed a brief screening questionnaire and then, if eligible, the survey.

Participants were able to enter a drawing to win one of three iPads on completion of the survey. No identifying information was collected, however, participants who chose to enter the drawing and/or request a copy of the study results were redirected to a separate survey form to enter their email address.

### 2.3. Measures

A Qualtrics survey was developed encompassing the Depression Anxiety Stress Scales (DASS-21 [25]) and a series of items designed for the purpose of this study, including a demographics questionnaire.

#### 2.3.1. Mental Health Measures

The DASS-21 [25] is a self-report questionnaire measuring negative emotional states of depression, anxiety and stress experienced over the past two-week period. It contains 21-items measuring three subscales (Depression, Anxiety, and Stress; seven items per subscale). Participants are asked to rate each item on a scale of 0 (did not apply to me at all) to 3 (applied to me very much). Total and subscale scores are computed by totalling the relevant items and multiplying the total by 2 (to enable comparison with normative data of the DASS-42). As such, the range of possible scores is 0–120 for the total score and 0–42 for the subscales. DASS-21 subscale scores can be assigned severity labels of “normal”, “mild”, moderate”, “severe”, and “extremely severe”. The DASS-21 has demonstrated good psychometric properties in samples of young people [26]. The internal consistency of each of the subscales in the current sample was excellent for depression (α = 0.93) and good for anxiety (α = 0.86) and stress (α = 0.87).

Participants were also asked if they had ever been diagnosed with a mental health problem, and to specify any diagnoses via open text response. Finally, participants were asked to use a 7-point Likert scale to rate their perceived impact of COVID-19 on their mental health, from “very negative”, “negative”, “somewhat negative”, “none at all”, “somewhat positive”, “positive”, and “very positive”. For ease of interpretation, these categories were collapsed into “negative impact”, “no impact”, and “positive impact”.

#### 2.3.2. Social Media Measures

A series of measures designed for the current study was used to assess social media use, both in general and for supporting suicidal thoughts and/or self-harm. Specifically, participants were asked to indicate how much time in a day and a week they spent using social media, which platforms they used and how often, and whether their social media use had changed during the COVID-19 pandemic. Next, participants were asked whether they had ever used social media to seek support for, or help others experiencing, self-harm or suicidal thoughts, and indicate which platforms were used for this purpose. Participants were asked about the last time they used social media to seek support or support someone else experiencing self-harm or suicidal thoughts, and to indicate how confident they felt doing this, and how they felt afterwards. Finally, they were asked about any social media platforms they perceived to be particularly unhelpful or harmful for talking about suicidal thoughts or self-harm. The majority of items were multiple-choice, with a small number of items inviting free-text responses.

### 2.4. Data Analysis

Free text responses relating to previous mental health diagnoses were coded as mood disorders (e.g., depression, bipolar disorder), anxiety disorders (e.g., generalised anxiety disorder, social anxiety disorder, or obsessive-compulsive disorder), eating disorders, personality disorders, trauma-related disorders (e.g., PTSD, complex PTSD), and other disorders (including autism spectrum disorder). Means and standard deviations of DASS-21 subscale and total scores, and frequencies of severity categories, were computed. Chi-square tests of independence or one-way ANOVAs with post hoc analyses, as appropriate, were used to examine the relationship between variables of DASS-21 subscale scores, mental health diagnoses, gender, and daily hours of social media use. To examine the differences between genders, the categories of “transgender”, “non-binary”, “other”, “unsure”, and “prefer not to say” were collapsed into a single category labelled “gender-diverse”. To analyse age differences, the sample was split into “older” and “younger” participants using the median age (22), with those aged 22 included in the “older” group. The remaining data were analysed using frequencies and percentages.

### 2.5. Ethics and Safety

This study was approved by the Melbourne University Human Research Ethics Committee (ID2056793). All participants provided informed consent prior to participating. Additionally, a list of crisis counselling telephone numbers and supportive websites was provided in the consent form and at the end of the survey.

## 3. Results

### 3.1. Participants

Of 599 young people who consented to participate and completed the screening questions, 8.2% (49/599) were excluded based on age (i.e., being younger than 16 or older than 25), 7.9% (47/599) were excluded because they said they lived outside of Australia, and 0.7% (4/599) were excluded for meeting both criteria (were either younger than 16 or older than 25 and lived outside Australia). Of the remaining 499 participants, 128 were excluded due to incomplete responses (defined as not completing at least the full DASS-21), leaving 371 participants with data amenable to analysis. As items did not force responses, not all participants responded to each question; therefore, the total number who responded (where known) is reported alongside the percentages.

Demographic information is presented in Table 1. The majority of participants were female (70.4%, 261/367), non-indigenous (97.0%, 356/367), from Victoria (68.0%, 249/366), and lived in a major city (59.5%, 217/365). Regarding education and employment status, 69.5% (258/371) were full-time or part-time students, and 51.2% (190/371) were employed in paid employment. Of the participants who stated they were currently unemployed, 74.5% (38/51) said they were currently seeking work, study, or both.

### 3.2. Mental Health

About half the sample (52.0%, 186/358) reported that they had been diagnosed with at least one mental health condition in their lifetime. These participants were invited to enter their diagnosis (or diagnoses if they had more than one). The most common diagnoses reported by participants were anxiety (79.6%, 148/186) or mood (71.0%, 132/186) disorders, followed by trauma-related (12.4%, 23/186), eating (8.1%, 15/186), and personality disorders (7.0%, 13/186). 

Table 2 presents Chi-square tests of independence examining the relationships between age, gender, and categorical variables. A significantly higher proportion of gender-diverse participants reported a diagnosis of a mental disorder (81.5%), compared with female participants (55.3%) and male participants (31.2%). A significantly higher proportion of older (58.4%) than younger participants (44.6%) also reported a diagnosis.

Total DASS-21 scores ranged from 0 to 126, with a mean score of 52.1 (*SD* = 28.8). Mean scores, standard deviations, and proportion of participants scoring in each severity category for each subscale are displayed in Table 3. A minority of participants scored in the “Normal” range for each variable; 41.8% of the sample (155/371) scored in the “Severe” or “Extremely Severe” range for depression, 41.0% (152/371) for anxiety, and 30.5% (113/371) for stress.

There were significant differences in DASS-21 subscale scores between genders (Table 4). A Tukey post hoc analysis revealed that participants identifying as gender-diverse scored significantly higher on the depression and anxiety subscales than both males and females (*p* = 0.000) and that there were no significant differences between males and females (*p* = 0.375 for depression; *p* = 0.147 for anxiety). For the stress subscale, a Games-Howell post hoc analysis showed that there were significant differences between all three groups, with participants identifying as gender-diverse scoring significantly higher than both males (*p* = 0.000) and females (*p* = 0.003), and female participants scoring higher than males (*p* = 0.015). There were significant differences between older and younger participants for the Anxiety subscale only, with younger participants returning higher scores than older participants (*p* = 0.020).

The majority of participants perceived COVID-19 to have had a negative impact on their mental health (86.0%, 308/358), 8.4% perceived no impact (30/358), and 5.5% perceived it to have had a positive impact (20/358). Chi-squared tests of independence revealed a significant association between having a diagnosed mental health condition and the perceived impact of COVID-19 on mental health, *X*^2^ (2, *n* = 350) = 14.549, *p* = 0.001. Although the majority in each category rated COVID-19 as having a negative impact on their mental health, a higher proportion of people with a mental health diagnosis rated the pandemic as having a negative impact on their mental health (91.1%) and a lower proportion reported the pandemic had no impact (2.8%) when compared to the group with no diagnosed mental health condition (80.7% and 14.0%, respectively). Additionally, Fisher’s exact test revealed there was also a significant association between gender and the perceived impact of COVID-19 on mental health (*p* = 0.009), with more females reporting a negative impact than males (87.8% vs. 74.7%) and all gender-diverse participants reporting a negative impact. There was no significant age difference for the perceived impact of COVID-19 (*p* = 0.256). See Table 2.

### 3.3. Social Media Use—General

The frequency of social media use is displayed in Table 5. The vast majority of participants (96.1%) reported using social media every day, with Instagram and Facebook the most commonly-used platforms on a daily basis. In an average day, almost half (46.2%, 164/355) used social media for 3–4 h, 21.7% (77/355) for 0–2 h, 15.5% (55/355) for 5–7 h, 6.8% (24/355) for less than one hour, 6.5% (23/355) for 7–10 h, and 3.4% (12/355) for more than 10 h.

There were significant age differences in daily hours of social media use, with younger participants spending more daily hours using social media than older participants. There were also gender differences in daily hours of social media use, with gender-diverse participants using social media most often, followed by females and then males (Table 2).

Two-thirds of participants (65.9%; 218/331) reported using social media more since the beginning of the COVID-19 pandemic, with 32.0% (106/331) using social media much more than before and 33.8% (112/331) a bit more than before. Most of the remaining reported using social media the same as before (25.1%; 83/331), 6.3% (21/331) less than before, and 2.7% (9/331) much less than before.

### 3.4. Associations between Social Media Use and DASS-21 Scores

One-way ANOVAs demonstrated that the DASS-21 subscale scores increased with increasing daily hours of social media use (Table 4). Tukey post hoc analyses revealed that DASS-21 depression scores were significantly higher in those who used social media for more than 7 h, compared to all other groups, and that anxiety scores were higher in those who used social media for more than 7 h, compared to those who used it for 0–2 or 3–4 h. For stress scores, a Games-Howell post hoc analysis revealed that scores were higher in those who used social media for more than 7 h, compared to those who used it for 0–2 or 3–4 h.

### 3.5. Social Media Use for Support with Suicidal Thoughts or Self-Harm

Participants were asked if they had ever used social media to deal with their own suicidal thoughts or self-harm or to help another person with suicidal thoughts or self-harm (note that these categories are not mutually exclusive). Thirty-six percent (125/347) reported that they had used social media to support themselves and 50.4% (172/341) had used it to support others. Chi-squared tests of independence revealed significant age and gender differences (see Table 2). For gender, 22.1% of male, 34.8% of female, and 82.1% of gender-diverse participants had used social media to help them deal with their own suicidal thoughts (*p* = 0.000), whereas 41.8% of male, 50.6% of female, and 71.4% of gender-diverse participants had used social media to support others (*p* = 0.031). In other words, gender-diverse young people were more likely than their cisgender peers to use social media in seeking or providing support for suicidal thoughts or self-harm. While a significantly higher proportion of younger participants (42.4%) than older participants (30.2%) had used social media to seek support, no significant age differences were identified for supporting others (Table 2).

Table 6 shows which social media sites were used for self-harm or suicide-related purposes. As can be seen, the most popular social media platform used for seeking support was YouTube (52.0%, 65/125), followed by Instagram (47.2%, 59/125) and Facebook (40.8%, 51/125). For supporting others, Facebook was by far the most popular platform (61.0%, 105/172), followed by Instagram (39.5%, 68/172). In terms of confidence, about half the participants reported feeling either “somewhat” or “fairly” confident using social media to seek support (50.4%, 63/125) and to support others (47.1%, 81/172). Participants were also asked about how they felt after the last time they used social media for this purpose; of those who had used social media to seek support, 67.9% (85/125) reported they felt better, 20.0% (25/125) felt the same, and 5.6% (7/125) felt worse, with one participant reporting they felt suicidal. After supporting others, 50.5% (87/172) reported they felt better, 20.9% (36/172) felt the same, and 25.0% (43/172) felt worse, with four reporting they felt suicidal.

Participants were asked if they had encountered any specific social media sites they would consider to be unhelpful or harmful when it comes to talking about self-harm and/or suicidal behaviour; 43.2% (51/118) indicated they had. Participants were invited to list those sites they believed to be unhelpful or harmful, of which 88.2% (45) responded. The following platforms were named by participants, in order of frequency of occurrence: Tumblr (*n* = 18), Facebook and Instagram (*n* = 8), Twitter and Reddit (*n* = 5), Tik Tok and Snapchat (*n* = 3), 4chan (an anonymous image-based bulletin board website; *n* = 2), and YouTube and Pinterest (*n* = 1).

## 4. Discussion

The aim of this study was to examine both the mental health and social media use of young Australians during the COVID-19 pandemic. To achieve these aims, an anonymous survey of a community-based sample of young Australians, recruited via social media, was conducted. The results indicate that levels of emotional distress were relatively high, with over 40% of the sample reporting severe or very severe levels of anxiety or depression; a figure highest amongst gender-diverse individuals. Further, 81% of young people without a mental health diagnosis and 91% of those with a diagnosis perceived the pandemic to have had a negative impact on their mental health. Young people reported using social media sites frequently, with 96% reporting use at least once a day, and two-thirds reporting that their usage of social media had increased as a result of the pandemic. There was a relationship between levels of psychological distress and the number of hours of daily social media use, with more severe emotional distress reported by those who used social media for more than 7 h per day. Furthermore, about one-third of those surveyed had used social media to seek support for suicidal thoughts or self-harm, and half had used it to support another person.

The levels of depression, anxiety, and stress in the sample were much higher than would be expected based on normative data and other validation studies of the DASS-21 with adolescent populations [25,26,27]. Psychological distress and perceptions of COVID-19 as having a negative impact on mental health were highest in gender-diverse participants, consistent with research suggesting that trans and gender-diverse young people are more vulnerable to the mental health impacts of the pandemic than their cisgender counterparts [28]. Additionally, in line with previous research, females reported higher levels of stress and more negative perceptions of the impact of the pandemic than males [29,30]. Taken together, the findings suggest that the COVID-19 pandemic may be associated with high levels of psychological distress, a finding that has been reported in an increasing number of published studies e.g., [2,3,4,31]. Interestingly, however, the DASS-21 scores in the present study were higher than those reported in other comparable studies examining the impact of COVID-19 on young people [32,33]. It should be noted that there was considerable variability in the sample, indicated by large standard deviations of DASS-21 subscale scores. This suggests that the negative impact varies between individuals and that there may be particular subgroups more or less vulnerable. Subgroups more vulnerable and likely over-represented in the current sample (when compared to other similar studies) include gender-diverse young people and those with a previous mental health diagnosis. Indeed, epidemiological data have indicated that one in seven young people meet the criteria for a mental health disorder [34], compared to half reporting a mental health diagnosis in the current sample. Participants in the current sample with an underlying mental health condition perceived the COVID-19 pandemic to have had a worse impact on their mental health than those without. This aligns with some literature suggesting that individuals with pre-existing mental health concerns may be more vulnerable to the impact of COVID-19-related stressors [5]. However, the perceived impact of COVID-19 does not necessarily equal actual impact: one study found that although people with an underlying mental health condition rated the pandemic as having a worse impact on their mental health than people without an underlying diagnosis, longitudinal data indicated they did not experience worsening of symptoms [35].

With regard to social media use, nearly all participants (96%) reported using social media at least once per day, with just under half the sample spending between 3 and 4 h per day on social media. Instagram and Facebook were the most commonly used social media sites, with about 70% of the sample using these platforms every day. Given research suggests that time spent online tends to be under-reported [36,37], these figures are likely to be an under-representation of daily hours spent using social media. Notably, two-thirds of participants reported they used social media more than they did before the COVID-19 pandemic, indicating social media may play a particularly important role in the lives of young people during this time. In line with previous research [38,39], heavy social media use (more than 7 h per day) was significantly associated with higher levels of depression, anxiety, and stress. Whilst it was beyond the scope of this study to assume causality or to explore whether characteristics of social media use (e.g., active vs. passive; quality of interactions) were associated with mental health outcomes, previous literature points to several possible explanations. First, individual factors such as inadequate offline social support or baseline social anxiety may explain associations between high levels of social media use and elevated mental health symptoms [40,41]. Second, young people may actively or passively view large volumes of information about the COVID-19 pandemic on social media, leading to a sense of “information overload” and subsequent increases in anxiety or distress [42]. A third possibility is young people with more severe mental health symptoms who are unable or unwilling to access mental health services during the pandemic (e.g., due to long waitlists) use social media frequently to seek help or receive support. As results suggest that the rates of mental distress are higher in this sample, possibly due to increased stress as a result of COVID-19, young people may be experiencing an increased need for mental health support and turning to social media in the absence of adequate alternatives.

This possibility may be supported by our finding that just over one-third of respondents had used social media to seek support for themselves and half had used it to support someone else; this finding adds to previous literature suggesting that young people do use social media to talk about suicide [20,24]. Significant gender differences were identified, with 82% and 71% of gender-diverse participants using social media to seek support and support others, respectively, compared to 35% and 51% of females and 22% and 42% of males. This is perhaps unsurprising given the high rates of suicidal ideation and behaviour in gender-diverse individuals [43], and relatedly, their high levels of exposure to suicide in their social networks [44]. Facebook and Instagram rated highly for both receiving and giving support, possibly due to their direct messaging capabilities which have previously been demonstrated to be popular among young people [45,46,47]. YouTube, however, was the most popular platform used to seek support; this is an interesting finding given that YouTube does not have a direct messaging function. The preference for seeking support on YouTube is concerning since the quality and safety of YouTube videos is questionable: one study found that very few YouTube videos discussing suicide-related content followed the safe and effective messaging guidelines established by the Suicide Prevention Resource Centre [48].

Despite the relatively high rates of social media use to seek and provide support, confidence levels were low. The majority of young people reported feeling at most “somewhat confident” in seeking support for themselves (59.2%) or supporting another person (66.9%) on social media. In terms of the impact of seeking help or supporting another person, the majority of participants reported feeling better afterwards and about one-fifth reported no change. This supports other studies that suggest those who seek help for self-harm via social media are likely to be met with positive reactions by their peers, such as offering help, receiving empathy, or connecting with others who understand what they are going through [49,50]. Far fewer participants reported negative outcomes: five percent of people who had sought help for suicidal thoughts or feelings using social media reported this made them feel worse, including suicidal. Interestingly, however, one-quarter of people who had supported somebody else with suicidal thoughts or feelings via social media said they felt worse afterwards, with four respondents indicating that they felt suicidal after doing so. These findings suggest that while seeking support for suicidal thoughts and feelings may be largely safe and acceptable, supporting others can have negative consequences for the helper. The heavy responsibility felt by young people and the challenges associated with providing online support has been documented and it is understood that viewing another person’s distress can impact the viewer’s own mental health [50]. Further, exposure to suicide-related content has been associated with an increased risk of suicidal ideation and attempts in young people [51]. Therefore, more research is required to understand what sort of interactions might lead to particularly negative or positive outcomes in these circumstances, including whether the attributes of the person providing support, such as their own lived experience of suicide risk and/or their wellbeing at baseline, have an impact. Taken together, these results emphasise the need to educate people about how to use social media safely and effectively to seek support for suicidal thoughts and feelings and to support others. The results also have implications for social media companies, which have a responsibility to be aware of the vulnerability of some users and take reasonable steps to promote safety. Tools such as the #chatsafe guidelines and accompanying social media campaign [52,53,54] may have an important role here and speak to the utility of providing mental health support to young people entirely via social media [54].

### Limitations

Several limitations of this study should be noted. First and foremost, data were obtained from a convenience sample of young people who self-selected into the study after viewing advertisements posted on social media (specifically, Facebook). Although paid Facebook advertising was used to maximise reach, this method of survey promotion together with the clearly-labelled survey purpose limits participation to those who already use Facebook and who may have a vested interest in the mental health impacts of the pandemic (e.g., due to personal experience), thus biasing the sample. The second limitation relates to the homogenous nature of the sample: the majority of participants were female, non-indigenous students from Victoria. Although this in part reflects the targeted recruitment efforts in Victoria (which has a lower percentage of Indigenous Australians compared to the national average), as well as data suggesting young females experienced higher rates of suicide attempts and self-harm in 2020 compared to males [55], it is acknowledged that results may not generalise beyond this sub-group of the Australian population. Third, the cross-sectional nature of the survey and lack of any pre-pandemic baseline data for this sample means that no conclusions can be drawn regarding causality. Fourth, it was beyond the scope of the present study to explore the nature or function of social media use, preventing speculation regarding the relationship between social media use and mental health or the quality of suicide prevention content on social media; further research in this area is therefore indicated. Fifth, the self-report nature of data collection means data are based on participants’ perceptions of their experiences and may lack objectivity. Sixth, participants were not asked about the nature of social support available to them outside of social media; it is acknowledged this may influence findings related to social media and should be a focus of future research in this area. Seventh, the dichotomisation of age into “older” and “younger” groups based on the median, whilst a common strategy in clinical research, has some inherent limitations, including reduced statistical power to detect differences between groups [56]. Finally, despite the focus of the latter component of this study on suicide and self-harm, we did not assess the prevalence of suicidality or self-harm in the sample. This was a conscious decision made due to ethical concerns associated with our inability to follow up with anyone indicating risk and limiting our ability to explore relationships between suicidality or self-harm, online help-seeking, and social media use.

## 5. Conclusions

Notwithstanding the limitations, this study provides valuable insights into mental health and the use of social media in young Australians during the COVID-19 pandemic. Psychological distress appears to be elevated during the COVID-19 pandemic, and young people generally perceive the pandemic to have had a negative impact on their mental health. Findings suggest that young people also spend more time on social media as a result of the pandemic, with high levels of daily social media use associated with poorer mental health. Further, young people also use social media to seek support for suicidal thoughts and self-harm, as well as to provide support to others; taken together this suggests social media may provide an opportunity to support young people experiencing psychological distress and suicide risk. Despite this, questions remain around the quality and safety of the types of support accessed by young people on social media; moreover, providing support to others may be associated with negative outcomes for some. Based on the findings of this study, we make the following key recommendations: (1) high-quality resources for both help-seekers and help-givers should be developed and disseminated to ensure young people have access to adequate support during the current COVID-19 pandemic and in the future; (2) efforts should be made to target particularly vulnerable groups, including young people with an underlying mental health condition and gender-diverse young people; and (3) social media companies should remain cognisant of the vulnerability of some users during crises such as pandemics, and do what they can to promote wellbeing and safety.

## Figures and Tables

**Table 1:** Demographic characteristics of the sample.

Characteristic	Response	*n* (%)
**Age, M (SD)**		21.1 (3.0)
**Gender**	Male	20.8 (77)
	Female	70.4 (261)
	Transgender	0.3 (1)
	Non-binary	4.1 (15)
	Other	1.1 (4)
	Unsure	1.9 (7)
	Prefer not to say	0.5 (2)
**Indigenous status**	Aboriginal	2.2 (8)
	Torres Strait Islander	0 (0)
	Both	0 (0)
	Prefer not to say	0.8 (3)
	Neither	97.0 (356)
**State**	Victoria	68.0 (249)
	New South Wales	11.5 (42)
	Tasmania	6.0 (22)
	Queensland	5.7 (21)
	Western Australia	3.0 (11)
	South Australia	2.7 (10)
	Australian Capital Territory	2.7 (10)
	Northern Territory	0.3 (1)
Area of residence *	Major City	59.5 (217)
	Inner regional	21.4 (78)
	Outer regional	10.7 (39)
	Remote	3.8 (14)
	Unsure	4.7 (17)
Education and employment **	Full-time student	58.2 (216)
	Part-time student	11.3 (42)
	Full-time employed	17.3 (64)
	Part-time employed	34.0 (126)
	Unpaid worker as parent or carer	1.6 (6)
	Unemployed *Seeking work* *Seeking study* *Seeking both*	13.7 (51) *43.1 (22)* *3.9 (2)* *27.5 (14)*

Note. M = Mean; SD = Standard Deviation. Italics denote responses to questions answered only those participants who indicated they were unemployed. Due to missing data, the total number of participants responding to each question is less than 371 (see Section 3.1). * Based on participant selection from a list of descriptors ** Categories not exclusive.

**Table 2:** Chi-square tests of independence examining gender and age differences for categorical variables.

	Gender					Age % (*n*)			
Characteristic	Male % (*n*)	Female % (*n*)	Gender- Diverse % (*n*)	Test Statistic	*p* Value	Younger % (*n*)	Older % (*n*)	Test Statistic	*p* Value
**Previous mental health diagnosis**				23.919	0.000			6.781	0.011
Yes	31.2 (24)	55.3 (140)	81.5 (22)			44.6 (75)	58.4 (111)		
No	68.8 (52)	44.7 (113)	18.5 (5)			55.4 (93)	41.6 (79)		
**Perceived impact of COVID-19**				NA *	0.009			2.887	0.256
Positive	8.0 (6)	5.5 (14)	0			9 (5.2)	11 (6.0)		
Negative	74.7 (56)	87.8 (224)	100.0 (27)			146 (83.9)	162 (88.0)		
Neutral	17.3 (13)	6.7 (17)	0			19 (10.9)	11 (6.0)		
**Hours of daily social media use**				NA *	0.005			12.139	0.007
0–2	42.5 (31)	25.7 (65)	14.3 (4)			21.6 (37)	34.8 (64)		
3–4	43.8 (32)	47.8 (121)	39.3 (11)			46.8 (80)	45.7 (84)		
5–7	9.6 (7)	16.6 (42)	21.4 (6)			17.5 (30)	13.6 (25)		
7+	4.1 (3)	9.9 (25)	25.0 (7)			14.0 (24)	6.0 (11)		
**Used social media to support self**				31.716	0.000			5.593	0.019
Yes	22.1 (15)	34.8 (87)	82.1 (23)			42.4 (70)	30.2 (55)		
No	77.9 (53)	65.2 (163)	17.9 (5)			57.6 (95)	69.8 (127)		
**Used social media to support others**				6.939	0.031			3.231	0.083
Yes	41.8 (28)	50.6 (124)	71.4 (30)			55.6 (90)	45.8 (82)		
No	58.2 (39)	49.4 (121)	28.6 (8)			44.4 (72)	54.2 (97)		

* Fisher’s exact test was conducted due to small group sizes.

**Table 3:** Proportion (%) of participants scoring in DASS-21 severity categories (*n* = 371).

DASS-21 Subscale	M (SD)	Normal	Mild	Moderate	Severe	Extremely Severe
**Depression**	19.2 (12.1)	23.2 (86)	14.3 (53)	20.8 (77)	13.5 (50)	28.3 (105)
**Anxiety**	13.5 (9.8)	29.4 (109)	15.9 (59)	13.7 (51)	14.0 (52)	27.0 (100)
**Stress**	19.4 (10.1)	37.2 (138)	12.7 (47)	19.7 (73)	18.9 (70)	11.6 (43)

**Table 4:** One-way ANOVAs exploring the relationship between DASS-21 subscale scores and gender, age, diagnosis, and social media use.

Variables	Depression						Anxiety						Stress					
	M	SD	F	df	*p*	η^2^	M	SD	F	df	*p*	η^2^	M	SD	F *	df	*p*	η^2^
**Gender**			9.315	2, 364	0.000	0.049			7.270	2, 364	0.001	0.038			12.252	2, 70	0.000	0.060
Male	17.0	12.6					11.2	10.0					15.9	10.7				
Female	19.0	11.7					13.5	9.4					19.7	9.7				
Gender-diverse	27.9	10.7					19.2	10.3					26.1	8.8				
**Age**			2.026	1, 369	0.155	-			5.489	1, 369	0.020	0.015			0.324	1, 369	0.569	-
Younger	20.2	12.1					14.7	9.4					19.7	9.7				
Older	18.4	12.1					12.4	10.0					19.1	10.7				
**MH diagnosis**			33.028	1, 356	0.000	0.085			49.034	1, 356	0.000	0.121			54.326	1, 354	0.000	0.132
Yes	22.5	12.0					16.6	9.8					22.9	9.6				
No	15.5	11.1					9.8	8.6					15.5	9.5				
**Daily SM use**			7.081	3, 351	0.000	0.057			6.069	3, 351	0.000	0.049			5.785	3, 115	0.001	0.043
0–2 h	16.3	11.8					11.0	10.1					17.3	11.3				
3–4 h	19.5	12.0					13.6	9.1					19.4	9.4				
5–7 h	19.8	11.8					14.1	9.2					20.8	9.9				
>7 h	26.9	11.3					18.9	10.2					24.8	8.6				

Note: SM = social media; * Welch’s F conducted for DASS-21 Stress scores as the assumption of homogeneity of variances was violated (*p* = 0.031).

**Table 5:** Frequency of use (% (*n*)) of different social media platforms (*N* = 360).

Social Media Platform	Daily	Weekly	Occasionally	Never
**Instagram**	70.8 (255)	13.9 (50)	8.3 (30)	6.9 (25)
**Facebook**	69.8 (251)	13.3 (48)	10.6 (38)	6.4 (23)
**Snapchat**	46.7 (168)	12.7 (45)	18.9 (68)	21.7 (78)
**YouTube**	45.6 (164)	33.6 (121)	14.7 (53)	6.1 (22)
**Tik Tok**	25.3 (91)	16.7 (60)	23.9 (86)	34.2 (123)
**Twitter**	17.2 (62)	12.2 (44)	36.4 (131)	34.2 (123)
**WhatsApp**	13.6 (49)	12.8 (46)	43.6 (157)	30.0 (108)
**Reddit**	11.2 (40)	11.2 (40)	43.3 (156)	34.4 (124)
**Interactive online games**	8.6 (31)	6.9 (25)	27.2 (98)	57.2 (206)
**LinkedIn**	4.4 (16)	12.5 (45)	46.1 (166)	36.9 (133)
**Tumblr**	4.2 (15)	9.1 (33)	40.3 (145)	46.4 (167)
**Pinterest**	3.9 (14)	14.6 (52)	46.4 (167)	35.3 (127)
**Weibo**	0.9 (3)	0.6 (2)	43.9 (158)	54.7 (197)
**Other**	3.3 (12)	2.7 (10)	23.1 (83)	70. 8 (255)
**All platforms (*N* = 363)**	96.1 (349)	1.9 (7)	1.2 (4)	0.8 (3)

Note: Due to missing data, the total number of participants responding to each question is less than 371 (see Section 3.1).

**Table 6:** Platform used, confidence and influence on mood % (*n*).

Variable		Supporting Self (*N* = 125)	Supporting Others (*N* = 172)
**Platform used**	YouTube	52.0 (65)	8.1 (14)
	Instagram	47.2 (59)	39.5 (68)
	Facebook	40.8 (51)	61.0 (105)
	Tumblr	24.0 (30)	13.4 (23)
	Snapchat	22.4 (28)	8.1 (14)
	Twitter	13.6 (17)	8.7 (15)
	Tik Tok	13.6 (17)	4.1 (7)
	Reddit	16.8 (20)	4.1 (7)
	Pinterest	9.6 (12)	0.6 (1)
	WhatsApp	0.8 (1)	4.1 (7)
	LinkedIn	0	0.6 (1)
**Confidence**	Not at all confident	13.6 (17)	11.0 (19)
	Slightly confident	19.2 (24)	32.6 (56)
	Somewhat confident	26.4 (33)	23.3 (40)
	Fairly Confident	24.0 (30)	23.8 (41)
	Completely confident	9.6 (12)	5.8 (10)
	No response	7.2 (9)	4.7 (8)
**Outcome**	Felt much better	12.8 (16)	17.4 (30)
	Felt a bit better	55.2 (69)	33.1 (57)
	Did not feel better or worse	20.0 (25)	20.9 (36)
	Felt a bit worse	4.0 (5)	16.3 (28)
	Felt much worse	0.8 (1)	6.4 (11)
	Felt suicidal	0.8 (1)	2.3 (4)
	No response	6.4 (8)	4.7 (8)

Note: *N* = number of participants reporting they had used social media to seek support (125) or support others (172).

## Data Availability

De-identified data can be provided on request.
